# Increased Platelet Reactivity Is Associated with Circulating Platelet-Monocyte Complexes and Macrophages in Human Atherosclerotic Plaques

**DOI:** 10.1371/journal.pone.0105019

**Published:** 2014-08-14

**Authors:** Bert Rutten, Claudia Tersteeg, Joyce E. P. Vrijenhoek, Thijs C. van Holten, Ellen H. A. M. Elsenberg, Elske M. Mak-Nienhuis, Gert Jan de Borst, J. Wouter Jukema, Nico H. J. Pijls, Johannes Waltenberger, Anton Jan van Zonneveld, Frans L. Moll, Elizabeth McClellan, Andrew Stubbs, Gerard Pasterkamp, Imo Hoefer, Philip G. de Groot, Mark Roest

**Affiliations:** 1 Laboratory of Clinical Chemistry and Hematology, University Medical Center Utrecht, Utrecht, the Netherlands; 2 Laboratory of Experimental Cardiology, University Medical Center Utrecht, Utrecht, the Netherlands; 3 Department of Vascular Surgery, University Medical Center Utrecht, Utrecht, the Netherlands; 4 Interuniversity Cardiology Institute of the Netherlands, Utrecht, the Netherlands; 5 Department of Cardiology, Leiden University Medical Center, Leiden, the Netherlands; 6 Department of Cardiology, Catharina Hospital, Eindhoven, the Netherlands; 7 Department for Cardiology, Maastricht University Medical Center, Maastricht, the Netherlands; 8 Department of Nephrology and Einthoven Laboratory for Experimental Vascular Medicine, Leiden University Medical Center, Leiden, the Netherlands; 9 Department of Bioinformatics, Erasmus University Medical Center, Rotterdam, the Netherlands; King’s College London School of Medicine, United Kingdom

## Abstract

**Objective:**

Platelet reactivity, platelet binding to monocytes and monocyte infiltration play a detrimental role in atherosclerotic plaque progression. We investigated whether platelet reactivity was associated with levels of circulating platelet-monocyte complexes (PMCs) and macrophages in human atherosclerotic carotid plaques.

**Methods:**

Platelet reactivity was determined by measuring platelet P-selectin expression after platelet stimulation with increasing concentrations of adenosine diphosphate (ADP), in two independent cohorts: the Circulating Cells cohort (n = 244) and the Athero-Express cohort (n = 91). Levels of PMCs were assessed by flow cytometry in blood samples of patients who were scheduled for percutaneous coronary intervention (Circulating Cells cohort). Monocyte infiltration was semi-quantitatively determined by histological examination of atherosclerotic carotid plaques collected during carotid endarterectomy (Athero-Express cohort).

**Results:**

We found increased platelet reactivity in patients with high PMCs as compared to patients with low PMCs (median (interquartile range): 4153 (1585–11267) area under the curve (AUC) vs. 9633 (3580–21565) AUC, P<0.001). Also, we observed increased platelet reactivity in patients with high macrophage levels in atherosclerotic plaques as compared to patients with low macrophage levels in atherosclerotic plaques (mean±SD; 8969±3485 AUC vs. 7020±3442 AUC, P = 0.02). All associations remained significant after adjustment for age, sex and use of drugs against platelet activation.

**Conclusion:**

Platelet reactivity towards ADP is associated with levels of PMCs and macrophages in human atherosclerotic carotid plaques.

## Introduction

Platelets and monocytes play a crucial role in the initiation and progression of atherosclerosis [1]. Platelets tether and roll over inflamed endothelial cells through transient interactions between platelet Glycoprotein Ibα (GPIbα) and endothelial P-selectin [2], [3]. The role of platelet binding to inflamed endothelial cells during the development of atherosclerosis was evidenced by a significant reduction in monocyte accumulation and atherosclerotic plaque progression after treatment with GPIbα blocking antibodies [4]. The interaction of platelets with inflamed endothelial cells also facilitates the capturing of monocytes to the vessel wall through interaction between Gp1bα and the constitutively expressed monocyte receptor P-Selectin Glycoprotein Ligand-1 (PSGL-1)[5]. This interaction, together with the interaction between GPIbα and the integrin macrophage-1 antigen (MAC-1), results in firm cellular arrest of monocytes and their subsequent infiltration into the vascular wall [6], [7]. Besides the capturing of monocytes to the vessel wall by interaction of monocytes with platelets that are bound to inflamed endothelial cells, platelets also bind circulating monocytes. Previous work has shown that formation of these circulating platelet-monocyte complexes results in increased infiltration of monocytes into atherosclerotic plaques in mice [8] and elevated levels of circulating PMCs were observed in patients with coronary artery disease [9]–[11].

Infiltration of monocytes into atherosclerotic plaques worsens atherosclerosis. Monocytes migrate into atherosclerotic plaques, where they differentiate into macrophages [12]. These macrophages secrete proteases and inflammatory proteins that weaken the fibrous cap, which increases the risk of plaque rupture. Rupture-prone plaques are characterized by a thin fibrous cap covering a large lipid core that is enriched with macrophages. Stable plaques contain less macrophages and are enriched with smooth muscle cells and high amounts of collagen [3]. After plaque rupture, platelets are activated and the formation of an occlusive thrombus can occur.

The role of platelet activation in atherosclerotic disease progression has been demonstrated by a reduction of cardiovascular events after platelet inhibition therapies [13]. Moreover, P-selectin knockout mice showed decreased monocyte adherence to the vessel wall and hence decreased atherosclerotic plaque formation [14], [15]. Platelet activation is triggered through stimulation of various receptors that stimulate integrin α_IIb_β_3_ activation, which is crucial for platelet aggregation. Several assays that measure platelet aggregation are currently available, e.g.; light transmission aggregometry (LTA), VerifyNow and platelet function analysis system (PFA-100). We have optimized a whole blood flow cytometry assay that measures platelet activation instead of platelet aggregation [16]. Our assay is based on the platelet response to increasing concentrations of adenosine diphosphate (ADP), which was quantified by the measurement of P-selectin upregulation after stimulation [17]. We used the platelet reactivity assay to investigate whether the reactivity of platelets towards ADP is associated with PMCs in circulation and macrophages in human atherosclerotic carotid plaques.

## Methods

### Ethics statement

All research in this study was approved by the ethics committees of the four participating medical centers in the Netherlands, being: Catharina Hospital in Eindhoven, University Medical Center in Maastricht, University Medical Center in Leiden and the University Medical Center in Utrecht. The study conforms to the Declaration of Helsinki and all participants provided written informed consent prior to participation.

### Study population

This report includes 2 cohorts: the Center for Translational Molecular Medicine (CTMM) cohort and the Athero Express (AE) cohort, executed at different laboratories in the Netherlands.

We analyzed 244 patients included in the CTMM cohort, who were scheduled for percutaneous coronary intervention, as previously described [18]. Exclusion criteria were age under 18 years, suspected alcohol or drug abuse, ST-elevation myocardial infarction, serious concomitant disease, an infection or suspected immune status elevation in six weeks prior to inclusion or absence of cooperation. Patients were included between September 2009 and April 2011.

We investigated 91 patients from the AE cohort, who were scheduled for carotid endarterectomy (CEA), as previously described [19]. Indications for CEA were reviewed by a multidisciplinary vascular team and were based on the recommended criteria of the Asymptomatic Carotid Atherosclerosis Study, the North American Symptomatic Carotid Endarterectomy Trial (NASCET: ISRCTN57874028), and the European Carotid Surgery Trial (ECST) [20], [21]. Patients undergoing CEA in the University Medical Center Utrecht were included between January 2011 and July 2012.

### Flow cytometry

Flow cytometry measurements were performed on the BD FACS Canto II (BD Biosciences). All samples were measured once. Compensation was calibrated using FITC and PE labeled beads according to the manufacturers protocol (BD Biosciences), and applied to all measurements. For the FITC signal, the solid state blue laser was used with a 530/30 filter. The PE signal was measured with the solid state blue laser and a 585/42 filter. Samples were measured using a flow rate of 1.5 µL/sec and data was acquired in a four-decade logarithmic scale. In the platelet activation assay, a gate was applied in the SSC-H/FSC-H plot around the GPIb^+^ (CD42b-FITC) cells. Within this gate, we measured 10.000 events. A blood sample that was not stimulated with a platelet agonist was used as a negative control to set the threshold for P-selectin (CD62P-PE) expression. With this threshold, we measured the increase in P-selectin median fluorescence intensity after stimulation with increasing concentrations of ADP. To measure PMCs, we gated CD14^+^ (CD14-PE) cells within the SSC-H/FSC-H plot and measured 15.000 events within this gate. Within this gate, the GPIb^+^ (CD42b-FITC) events were measured. An isotype control was used as a negative control for GPIb expression. Flow cytometry data was analyzed using FACS Diva software (BD Biosciences).

### Blood collection and platelet reactivity assay

Arterial blood samples were collected in citrate (3.2%) anticoagulated tubes (BD Biosciences) prior to intervention and processed directly after collection to prevent any potential blood storage effects.

**Figure 1:**
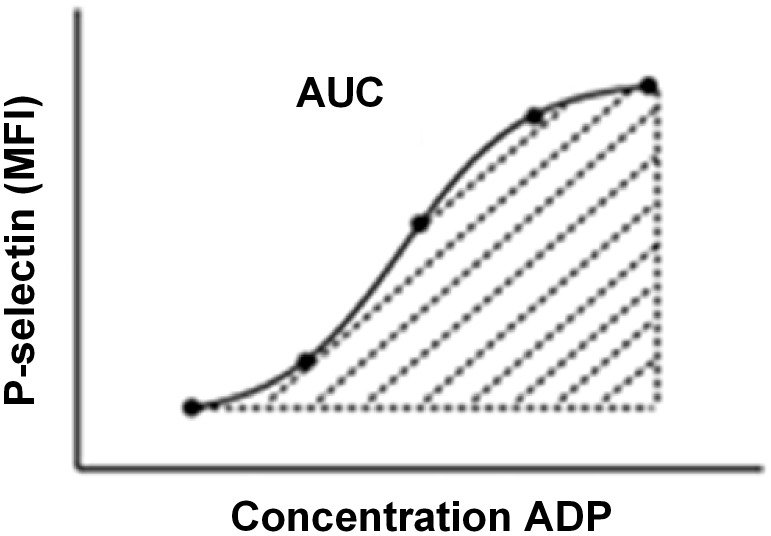
Conversion of dose-response curve into area under the curve. In order to compare total platelet reactivity between patients, we derived the area under the curve from each dose response curve. MFI = mean fluorescence intensity, AUC = area under curve, ADP = adenosine diphosphate.

Platelet reactivity was quantified by stimulation with an increasing concentration of ADP, ranging from 8 nM to 125 µM. The serial dilutions were prepared in 50 µL HEPES buffered saline (HBS; 10 mM HEPES, 150 mM NaCl, 1 mM MgSO_4_, 5 mM KCl, pH 7.4) to which 2 µL CD62P-PE (550561, BD Biosciences) and 2 µL CD42b-FITC (555472, BD Biosciences) mouse anti-human antibodies were added. The platelet reactivity assay was initiated by the addition of 5 µL whole blood to each sample of the serial dilution for 20 minutes at room temperature. Samples were fixated with 500 µL of 0.2% formaldehyde in 0.9% NaCl solution. Using flow cytometry, 10.000 platelets were identified by scatter gating and CD42b-FITC binding, after which CD62P-PE mean fluorescence intensity (MFI) was measured. We used the area under the curve (AUC) to integrate the sensitivity and the maximum response of platelets towards agonist concentrations into one quantitative value (Figure 1). All data acquisition was performed with the CXP SYSTEM software.

### Measurement of PMCs in the CTMM cohort

Circulating PMCs were quantified with a mixture of 40 µL HEPES buffered saline (HBS; 10 mM HEPES, 150 mM NaCl, 1 mM MgSO_4_, 5 mM KCl, pH 7.4) to which 5 µL CD14-PE (555398, BD Biosciences) and 5 µL CD42b-FITC (555472, BD Biosciences) mouse anti-human antibodies were added. Subsequently, 50 µL of whole blood was added to the mixture for 30 minutes at room temperature. Samples were fixated with 80 µL of Optilyse B (Beckman Coulter), containing 3.4% paraformaldehyde for 10 minutes, after which red blood cells were lysed by the addition of 825 µL of demineralized water. Monocytes were identified by scatter gating and CD14 labeling. PMCs were determined by the percentage of monocytes that were positive for the platelet marker CD42b-FITC. Fifteen thousand cells were counted on the same day of processing and data acquisition was performed with the CXP SYSTEM software.

### Histological examination of macrophages in atherosclerotic carotid plaques in the AE cohort

Atherosclerotic carotid plaques were collected during CEA and immediately transported to the laboratory for processing. Using a standardized protocol, plaques were divided in 5-mm segments along the longitudinal axis, followed by fixation with 4% paraformaldehyde and embedment in paraffin. The segment with the largest plaque burden was defined as the culprit lesion. Subsequent histological plaque assessment was performed by an experienced technician, blinded for patient characteristics or clinical outcome, with a good inter- and intraobserver variability [22]. Macrophages were identified by CD68 labeling and semi-quantitatively categorized in two categories, being low and high staining, at original magnification x40. Plaques were categorized as low macrophage staining if staining was negative or clusters with <10 cells were present. Cell clusters >10 cells or abundance of positive cells were categorized as high staining.

### Statistical analysis

Patients included in the CTMM cohort were divided into groups with low or high levels of PMCs based on the median value of these complexes. The CTMM platelet reactivity values were not normally distributed, therefore we used the non-parametric Mann-Whitney U test to compare platelet reactivity levels between patients in the low and high levels of PMCs groups. For the same reason, platelet reactivity values were transformed into natural logarithmic values and adjusted for age, sex and acetylsalicylic acid use in univariate analysis of variance. Since previous research demonstrated reduced platelet reactivity and PMC formation in patients treated with clopidogrel [23], [24], we performed a subanalysis after stratification on clopidogrel use.

Patients included in the AE cohort were divided into groups with low or high levels of macrophages in plaques based on semi-quantitative analysis. AE platelet reactivity values were normally distributed. Therefore, we used the parametric Student’s t-test to compare levels of platelet reactivity between patients in the low or high macrophage groups and logarithmic transformation was not necessary. Hereafter, AE platelet reactivity values were adjusted for age, sex, acetylsalicylic acid and clopidogrel use in univariate analysis of variance. Because 9 patients (10%) were treated with clopidogrel, of which only 1 patient had a plaque with a high level of macrophages, we did not separately assess clopidogrel-treated and non-treated patients.

All statistical analyses were performed using SPSS version 20.0 software (IBM Corp, IBM SPSS Statistics for Windows, Armonk, NY) and a two-sided P-value <0.05 was considered statistically significant.

## Results

### Baseline characteristics

The baseline characteristics and medication use at inclusion of patients investigated in the CTMM cohort (n = 244) and in the AE cohort (n = 91) are shown in table 1.

**Table 1:** Baseline characteristics.

	CTMM population	AE population
	(n = 244)	(n = 91)
Age in years	63±9.7	71.6±9.5
Male sex	167/244 (69%)	66/91 (73%)
Body mass index (kg/m^2^)	27.7±4.5	25.4±5.1
Smoking	53/244 (22%)	31/88 (35%)
Diabetes mellitus	45/244 (19%)	18/91 (20%)
Hypertension	157/244 (65%)	54/88 (61%)
Dyslipidemia	155/244 (64%)	53/75 (71%)
Total cholesterol (mmol/L)	4.4±1.2	4.9±1.5
History of coronary artery disease	115/244 (48%)	36/91 (40%)
Kidney disease	6/244 (3%)	NA
Glomerular Filtration Rate, CG, (mL/min)	NA	69.2±24.5
Acetylsalicylic acid	198/244 (83%)	73/89 (82%)
Clopidogrel	131/244 (55%)	9/89 (10%)
Dipyridamole	Not available	62/90 (69%)
Statins	192/244 (80%)	77/90 (86%)
Nitrates	88/244 (37%)	Not available
Clinical presentation	Not applicable	
* Asymptomatic*		14/89 (16%)
* Amaurosis fugax*		23/89 (26%)
* Transient ischemic attack*		26/89 (29%)
* Stroke*		26/89 (29%)
Bilateral carotid stenosis (>50%)	Not applicable	33/72 (47%)
Days from clinical event to CEA (median [IQR])	Not applicable	18 [11]–[31]

Continuous values are expressed as mean ± standard deviation (unless specified otherwise). Categorical values are expressed as number of total (percentage).

CG = Cockroft-Gault, CEA = carotid endarterectomy, IQR = interquartile range.

In the CTMM cohort, 189 patients (77.5%) were presenting with stable angina and 32 patients (13.3%) with unstable angina and 23 patients (9.5%) with nSTEMI. Subjects with stable angina had lower mean cholesterol levels (P = 0.03) and a higher prevalence of renal failure (P = 0.01) as compared with subjects with unstable angina, while subjects with nSTEMI used nitrates less frequently (P = 0.05) as compared with subjects with stable angina. CTMM patients were divided into groups with high (n = 122) and low levels of PMCs (n = 122) based on the median level of PMCs.

In the AE cohort, 75 patients (84%) had symptomatic carotid artery disease. AE patients were divided into groups with high (n = 24) and low (n = 67) levels of macrophages in the carotid plaques.

No significant differences were observed in baseline characteristics between patients in the high versus low groups within the CTMM and AE cohort. Moreover, no significant differences in baseline P-selectin expression before platelet stimulation with ADP were observed between the two groups within both cohorts (data not shown).

### High platelet reactivity is associated with increased levels of PMCs

**Figure 2:**
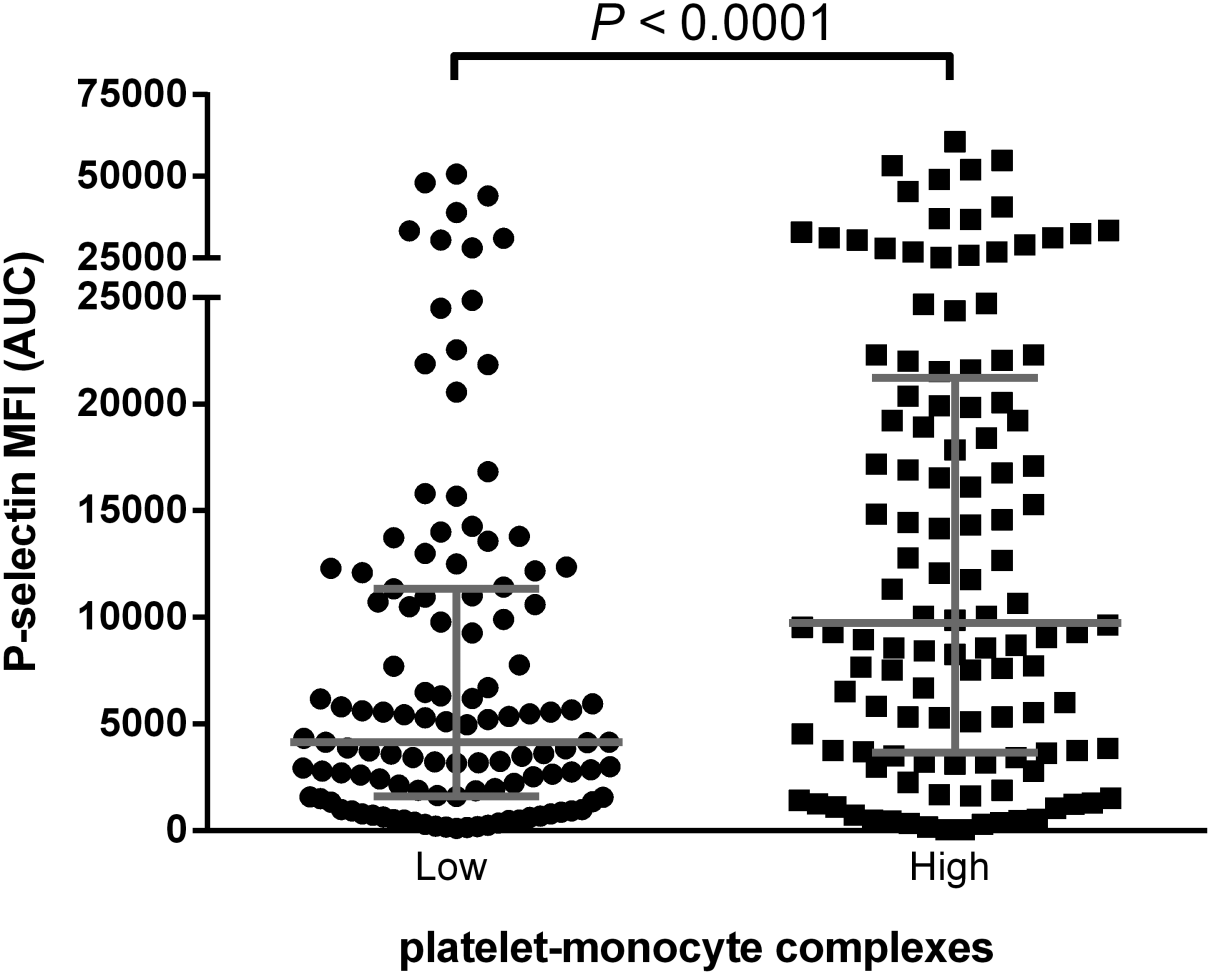
Platelet reactivity related to platelet-monocyte complexes. Comparison of the AUC after ADP stimulation between low (n = 122) versus high levels of platelet-monocyte complexes (n = 122). Grey bars represent medians with interquartile ranges. MFI = mean fluorescence intensity, AUC = area under curve, ADP = adenosine diphosphate.

In the CTMM cohort, we found a higher AUC after platelet stimulation with ADP in patients with high levels of PMCs as compared to patients with low levels of PMCs (median (interquartile range (IQR)); 9633 (3580–21565) AUC vs. 4153 (1585–11267) AUC, P<0.001) (Figure 2, Table S1). These associations remained significant after natural logarithmic transformation and adjustment for age, sex, acetylsalicylic acid and clopidogrel use (Table S1).

Next, we compared the AUC after platelet stimulation between patients not treated with clopidogrel to patients treated with clopidogrel. We found a higher AUC in patients not treated with clopidogrel (n = 109) as compared to patients treated with clopidogrel (n = 131) (median (IQR); 9784 (3206–21955) AUC vs. 5329 (866–11430) AUC, P = 0.001) (Table S2). Subsequently, we stratified patients on clopidogrel use and performed a sub analysis. In patients treated with clopidogrel, a higher AUC was found in patients with high levels of PMCs (n = 64) as compared to patients with low levels of PMCs (n = 67) (median (IQR); 2252 (1408–2784) AUC vs. 1447 (942–2329) AUC, P = 0.002) (Table S2). In patients not treated with clopidogrel, we also found a higher AUC in patients with high levels of PMCs (n = 57) as compared to patients with low levels of PMCs (n = 52) (median (IQR); 2878 (1866–3330) AUC vs. 1881 (1035–2793) AUC, P = 0.009) (Table S2).

Next, we adjusted for age, sex and acetylsalicylic acid. All associations between platelet reactivity and levels of PMCs remained significant in both clopidogrel-treated patients (mean±SD, 2051±933 AUC vs. 1550±933AUC, P = 0.003) and non-treated patients (mean±SD, 2521±1120 vs. 1945±1121, P = 0.009) (Table S2).

### High platelet reactivity is associated with high levels of macrophages in atherosclerotic carotid plaques

**Figure 3:**
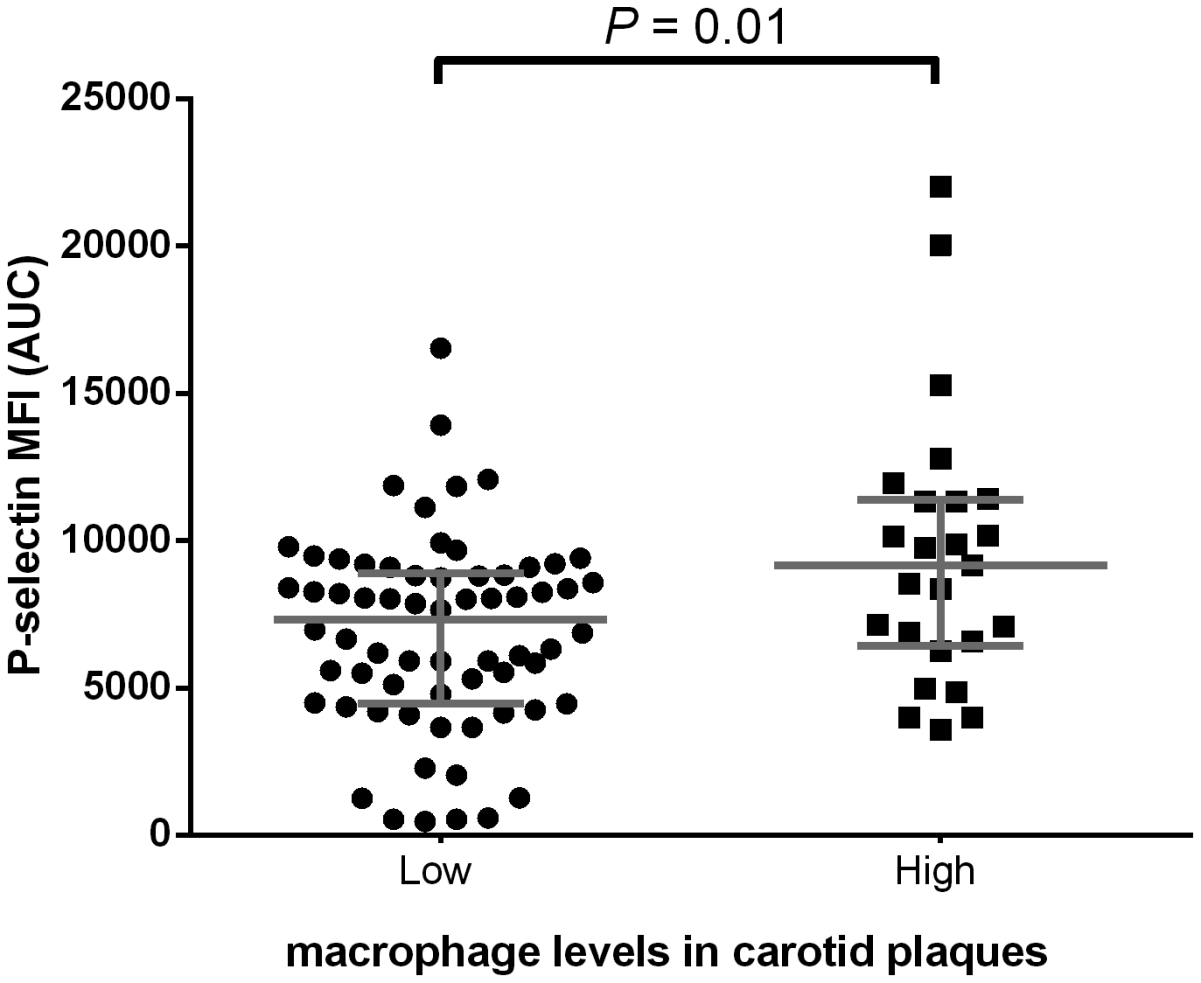
Platelet reactivity related to macrophage levels. Comparison of the AUC after ADP stimulation between low (n = 67) versus high macrophage levels in atherosclerotic carotid plaques (n = 24). Grey bars represent means with standard deviations. MFI = mean fluorescence intensity, AUC = area under curve, ADP = adenosine diphosphate.

In the AE cohort, we observed a higher AUC after platelet stimulation with ADP in patients with high levels of macrophages in atherosclerotic carotid plaques as compared to patients with low levels of macrophages in atherosclerotic carotid plaques (mean±SD, 9255±4530 AUC vs. 6995±3406 AUC, P = 0.01) (Figure 3, Table S3). Adjustment for age, sex, acetylsalicylic acid and clopidogrel use did not change the association between platelet reactivity and macrophages in atherosclerotic plaques (mean±SD, 8969±3485 vs. 7020±3442, P = 0.02) (Table S3).

**Figure 4:**
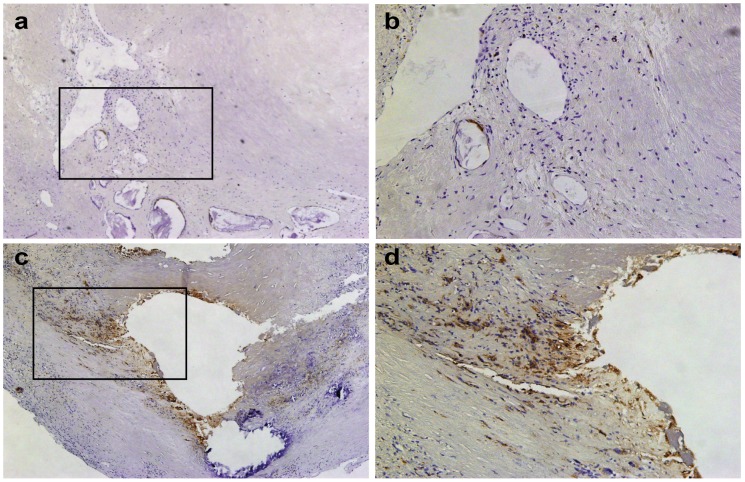
Histology of macrophage levels in atherosclerotic carotid plaques. Macrophage staining (brown), nucleus staining with haematoxylin (blue). (**a**) Low macrophage levels (40× magnification). (**b**) Higher magnification of the indicated area in (a) (100× magnification). (**c**) High macrophage levels (40× magnification). (**d**) Higher magnification of the indicated area in (c) (100× magnification).

Figure 4 shows representative images of carotid plaques with low (Figure 4a, with higher magnification in 4b) and high (Figure 4c, with higher magnification in 4d) amounts of macrophages in the plaque.

## Discussion

In patients referred for percutaneous coronary intervention, we found that high platelet reactivity was associated with high levels of circulating PMCs. In patients undergoing carotid endarterectomy, we observed that high platelet reactivity was associated with high levels of macrophages in atherosclerotic carotid plaques. All associations were found to be independent of age, sex and the use of drugs that inhibit platelet activation.

We determined platelet reactivity by measuring P-selectin upregulation after stimulation with increasing concentrations of ADP, using flow cytometry [17]. ADP activates platelets via the P2Y12 and P2Y1 receptors, which have been implicated as important mediators of atherosclerosis [25], [26]. Inflamed endothelium does not induce excessive platelet activation, and, similarly, ADP is a mild activator of platelet activation. Moreover, previous work from our department shows that measurement of platelet reactivity towards a mild activator like ADP is more likely to differ between cases and controls than stronger activators like thrombin receptor activating peptide (TRAP), collagen related peptide (CRP) or convulxin (a snake venom toxin that activates platelets) [27]. Hence, we feel that ADP is a suitable agonist that represents *in vivo* platelet stimulation. We used P-selectin as a marker of platelet activation, because P-selectin is translocated from the inner α-granule membrane to the outer platelet cell membrane after platelet activation. For this reason, P-selectin expression on the platelet cell membrane is currently the most commonly used marker of platelet activation.

The majority of commercially available platelet function assays, which have been questioned regarding their sensitivity and reproducibility [28]–[31], measure platelet aggregation. In contrast to these assays, we measure platelet reactivity. Evidence for a role of platelet reactivity in the progression of atherosclerosis was previously shown by infusion of activated platelets into ApoE knockout mice. The infused activated platelets adhered to monocytes, which resulted in increased atherosclerotic lesion size [8]. The mechanism by which platelets play a role in monocyte infiltration depends on the interaction between P-selectin on the platelet membrane and P-Selectin Glycoprotein Ligand-1 (PSGL-1) on the monocyte cell membrane. The binding of P-selectin to PSGL-1 induces the activation of integrins on the monocyte cell membrane, which results in enhanced adhesion of monocytes to inflamed endothelial cells [32], [33]. Also, the release of potent inflammatory proteins from the platelet α-granule during activation enhances the adhesive and chemotactic properties of endothelial cells [34], [35] and the release of monocyte chemoattractants, such as PF4 and RANTES, contributes to the infiltration of monocytes into atherosclerotic plaques [36]–[38]. The release of α-granule proteins is dependent on platelet reactivity towards agonists, but the relationship between platelet reactivity, PMC formation and macrophages in atherosclerotic carotid plaques has not yet been fully investigated. Previously it was shown that levels of circulating PMCs and percentages of platelets expressing P-selectin after stimulation were increased in 19 patients with stable angina compared to 19 healthy control subjects [10]. We extend these results by showing, in a population of 244 coronary artery disease patients, an independent association between high P-selectin expression after ADP stimulation and increased levels of circulating PMCs. Additionally, we show in 91 carotid endarterectomy patients that high P-selectin expression is associated with increased monocyte infiltration into atherosclerotic carotid plaques, independent of investigated covariates.

Clopidogrel, which blocks P2Y12 receptors, is used to prevent platelet aggregation in cardiovascular diseases. In the AE cohort, only 10% of the patients received clopidogrel, while, in the CTMM cohort, 55% of the patients received clopidogrel. Due to the P2Y12 block, platelets are less sensitive towards ADP stimulation. Affirmatively, we found a significant decrease in the AUC after platelet stimulation with ADP when clopidogrel users were compared with non-clopidogrel users in the CTMM cohort. However, we observed no significant differences in levels of PMCs when clopidogrel users were compared with non-clopidogrel users (data not shown). Importantly, even though clopidogrel use decreased platelet activation, the association between platelet reactivity and levels of PMCs remained significant. In the AE cohort, 9 patients received clopidogrel, of which only one had a plaque with high infiltration of macrophages. Hence, we could not analyze the effect of clopidogrel on levels of macrophages in plaques. Within both cohorts, no patients were treated with other P2Y12 inhibiting drugs, like cangrelor or prasugrel.

A limitation of this study is that levels of PMCs and levels of macrophages in atherosclerotic plaques were not assessed in both the CTMM cohort and the AE cohort, which included patients with different vascular diseases. Therefore we could not study whether platelet reactivity, levels of PMCs and levels of macrophages in atherosclerotic plaques were directly linked to each other. Nevertheless, the independent associations that we found in these two cohorts of patients with different vascular diseases, underscore the potential role of platelet reactivity in cardiovascular disease.

In conclusion, our observations show that increased platelet reactivity is independently associated with increased levels of circulating PMCs and macrophages in human atherosclerotic carotid plaques. This may suggest that decreased levels of circulating PMCs and macrophages in atherosclerotic plaques might be achieved by platelet inhibition.

## Supporting Information

Table S1 **Association of platelet reactivity with platelet-monocyte complexes (PMC).** All values are area under the curve after adenosine diphosphate stimulation and represent platelet reactivity. *Unadjusted values are before natural logarithmic transformation. **Adjusted values are after natural logarithmic transformation and are corrected for age, sex and acetylsalicylic acid and clopidogrel. †Comparison by Mann-Whitney U test. ‡ Comparison by univariate analysis of variance.
(DOCX)

Table S2 **Association of platelet reactivity with platelet-monocyte complexes (PMC) in clopidogrel treated and non-treated patients.** All values are area under the curve after adenosine diphosphate stimulation and represent platelet reactivity. *Unadjusted values are before natural logarithmic transformation. **Adjusted values are after natural logarithmic transformation and are corrected for age, sex and acetylsalicylic acid and clopidogrel. †Comparison by Mann-Whitney U test. ‡ Comparison by univariate analysis of variance.
(DOCX)

Table S3 **Association of platelet reactivity with macrophages in atherosclerotic plaques after.** All values are area under the curve after adenosine diphosphate stimulation and represent platelet reactivity. *Adjusted values are corrected for age, sex, acetylsalicylic acid and clopidogrel use. †Comparison by Student’s *t-*test. ‡Comparison by univariate analysis of variance.
(DOCX)
