# Supplementary material for: Increased Platelet Reactivity Is Associated with Circulating Platelet-Monocyte Complexes and Macrophages in Human Atherosclerotic Plaques
Source: PLoS One. 2014 Aug 14;9(8):e105019. doi: 10.1371/journal.pone.0105019 (PMC4133361; doi:10.1371/journal.pone.0105019)
Supplement: Table S1 — Association of platelet reactivity with platelet-monocyte complexes (PMC). All values are area under the curve after adenosine diphosphate stimulation and represent platelet reactivity. *Unadjusted values are before natural logarithmic transformation. **Adjusted values are after natural logarithmic transformation and are corrected for age, sex and acetylsalicylic acid and clopidogrel. †Comparison by Mann-Whitney U test. ‡ Comparison by univariate analysis of variance. (DOCX) [file pone.0105019.s001.docx]

|  | **Unadjusted*,  median (IQR)** | ***P*-value** | **Adjusted**, mean (SD)** | ***P*-value** |
| --- | --- | --- | --- | --- |
| **CTMM population** (n=244) | 6329 (2348-15955) |  | Not applicable |  |
| Low PMC (n=122) | 4153 (1585-11267) |  | 1729 (1015) |  |
| High PMC (n=122) | 9633 (3580-21565) | <0.001† | 2267 (1015) | <0.001‡ |
