# Supplementary material for: Increased Platelet Reactivity Is Associated with Circulating Platelet-Monocyte Complexes and Macrophages in Human Atherosclerotic Plaques
Source: PLoS One. 2014 Aug 14;9(8):e105019. doi: 10.1371/journal.pone.0105019 (PMC4133361; doi:10.1371/journal.pone.0105019)
Supplement: Table S2 — Association of platelet reactivity with platelet-monocyte complexes (PMC) in clopidogrel treated and non-treated patients. All values are area under the curve after adenosine diphosphate stimulation and represent platelet reactivity. *Unadjusted values are before natural logarithmic transformation. **Adjusted values are after natural logarithmic transformation and are corrected for age, sex and acetylsalicylic acid and clopidogrel. †Comparison by Mann-Whitney U test. ‡ Comparison by univariate analysis of variance. (DOCX) [file pone.0105019.s002.docx]

|  | **Unadjusted*,  median (IQR)** | ***P*-value** | **Adjusted**, mean (SD)** | ***P*-value** |
| --- | --- | --- | --- | --- |
| **CTMM population** (n=244) | 6329 (2348-15955) |  | Not applicable |  |
| Clopidogrel (-) (n=109) | 9784 (3206-21955) |  |  |  |
| Clopidogrel (+) (n=131) | 5329 (866-11430) | 0.001† |  |  |
| Clopidogrel (+) High PMC (n=64) | 2252 (1408-2784) |  | 2051 (933) |  |
| Clopidogrel (+) Low PMC (n=67) | 1447 (942-2329) | 0.002† | 1550 (933) | 0.003‡ |
| Clopidogrel (-) High PMC (n=57) | 2878 (1866-3330) |  | 2521 (1120) |  |
| Clopidogrel (-) Low PMC (n=52) | 1881 (1035-2793) | 0.009† | 1945 (1121) | 0.009‡ |
