# Supplementary material for: Increased Platelet Reactivity Is Associated with Circulating Platelet-Monocyte Complexes and Macrophages in Human Atherosclerotic Plaques
Source: PLoS One. 2014 Aug 14;9(8):e105019. doi: 10.1371/journal.pone.0105019 (PMC4133361; doi:10.1371/journal.pone.0105019)
Supplement: Table S3 — Association of platelet reactivity with macrophages in atherosclerotic plaques after. All values are area under the curve after adenosine diphosphate stimulation and represent platelet reactivity. *Adjusted values are corrected for age, sex, acetylsalicylic acid and clopidogrel use. †Comparison by Student’s t-test. ‡Comparison by univariate analysis of variance. (DOCX) [file pone.0105019.s003.docx]

|  | **Unadjusted,  mean (SD)** | ***P*-value** | **Adjusted*, mean (SD)** | ***P*-value** |
| --- | --- | --- | --- | --- |
| **Athero-Express population** (n=91) | 7591 (3841) |  | Not applicable |  |
| Low macrophages (n=67) | 6995 (3406) |  | 7020 (3442) |  |
| High macrophages (n=24) | 9255 (4530) | 0.01† | 8969 (3485) | 0.02‡ |
